# Development of Focused Ultrasound-Assisted Nanoplexes for RNA Delivery

**DOI:** 10.3390/nano14131089

**Published:** 2024-06-25

**Authors:** Sanjeev Ranjan, Stef Bosch, Hannamari Lukkari, Johanna Schirmer, Niina Aaltonen, Heikki J. Nieminen, Vesa-Pekka Lehto, Arto Urtti, Tatu Lajunen, Kirsi Rilla

**Affiliations:** 1 Institute of Biomedicine, University of Eastern Finland, 70210 Kuopio, Finland; sanjeev.ranjan@aalto.fi (S.R.); stef.bosch@uef.fi (S.B.); hannamari.lukkari@outlook.com (H.L.); niina.aaltonen@uef.fi (N.A.); 2 Medical Ultrasonics Laboratory (MEDUSA), Department of Neuroscience and Biomedical Engineering, Aalto University, 02150 Espoo, Finland; heikki.j.nieminen@aalto.fi; 3 FinVector Oy, 70210 Kuopio, Finland; 4 Nanoscience Center, Department of Chemistry, University of Jyväskylä, 40014 Jyväskylä, Finland; johanna.schirmer@outlook.com; 5 Department of Technical Physics, University of Eastern Finland, 70210 Kuopio, Finland; vesa-pekka.lehto@uef.fi; 6 School of Pharmacy, University of Eastern Finland, 70210 Kuopio, Finland; arto.urtti@uef.fi (A.U.); tatu.lajunen@uef.fi (T.L.); 7 Drug Research Program, Faculty of Pharmacy, University of Helsinki, 00100 Helsinki, Finland

**Keywords:** focused ultrasound, nanoplexes, siRNA delivery, phospholipid bilayer fragments, encapsulation efficiency, lipid nanoparticles

## Abstract

RNA-based therapeutics, including siRNA, have obtained recognition in recent years due to their potential to treat various chronic and rare diseases. However, there are still limitations to lipid-based drug delivery systems in the clinical use of RNA therapeutics due to the need for optimization in the design and the preparation process. In this study, we propose adaptive focused ultrasound (AFU) as a drug loading technique to protect RNA from degradation by encapsulating small RNA in nanoliposomes, which we term nanoplexes. The AFU method is non-invasive and isothermal, as nanoplexes are produced without direct contact with any external materials while maintaining precise temperature control according to the desired settings. The controllability of sample treatments can be effectively modulated, allowing for a wide range of ultrasound intensities to be applied. Importantly, the absence of co-solvents in the process eliminates the need for additional substances, thereby minimizing the potential for cross-contaminations. Since AFU is a non-invasive method, the entire process can be conducted under sterile conditions. A minimal volume (300 μL) is required for this process, and the treatment is speedy (10 min in this study). Our in vitro experiments with silencer CD44 siRNA, which performs as a model therapeutic drug in different mammalian cell lines, showed encouraging results (knockdown > 80%). To quantify gene silencing efficacy, we employed quantitative polymerase chain reaction (qPCR). Additionally, cryo-electron microscopy (cryo-EM) and atomic force microscopy (AFM) techniques were employed to capture images of nanoplexes. These images revealed the presence of individual nanoparticles measuring approximately 100–200 nm in contrast with the random distribution of clustered complexes observed in ultrasound-untreated samples of liposome nanoparticles and siRNA. AFU holds great potential as a standardized liposome processing and loading method because its process is fast, sterile, and does not require additional solvents.

## 1. Introduction

Lipid-based nanoparticles, such as liposomes, have gained prominence in drug and gene delivery due to their compact size, enhanced cellular uptake, prolonged action, and reduced systemic side effects. They have many other advantages, including their formulation simplicity, self-assembly-based production, ability to carry large payloads of nucleic acids, potential for cell-targeted drug delivery, and controllable physicochemical properties that can be used to modulate their biological characteristics. For these reasons, lipid-based nanoparticles are the most common class of FDA-approved nanomedicines [1,2]. However, despite these advantages, lipid-based nanoparticles face several limitations. These include potential instability during storage, susceptibility to environmental factors, challenges related to drug loading, and achieving high encapsulation efficiency for therapeutic agents [3,4]. To address these issues, further research is necessary to optimize liposome drug loading techniques. Additionally, a comprehensive understanding of their structural properties is crucial for refining their design and achieving improved therapeutic outcomes.

Classical lipid-based lipoplexes are based on electrostatic binding between cationic lipids and nucleic acids, such as DNA and RNA [5]. Even though lipoplexes can be conveniently produced, their preparation is often poorly controlled, leading to polydisperse particles with unpredictable and variable efficacy of transfection or silencing in target cells. More specifically, these classical production methods have drawbacks for nucleic acid delivery [1,2,3,4,6]. Non-uniform lipoplexes are formed due to the poorly controlled fusion of liposomes or other lipid vesicles with nucleic acids in solution [2]. This may lead to heterogenous interactions (electrostatic, hydrophobic, surface adsorption) and opsonization within the lipoplexes, resulting in non-reproducible interactions with target cells and extracellular components. The large and heterogeneous size of lipoplexes limits in vivo transfection due to poor escape from the vascular bed and sub-optimal endocytic entry to the target cells [1]. Unencapsulated nucleic acids are unstable in the body and vulnerable to degradation by nucleases [3]. The reticuloendothelial system rapidly clears large and heterogeneous lipoplexes that are also more susceptible to surface opsonization that tends to hamper targeted delivery to the sites of therapeutic action [4].

The method that is gaining lots of interest and currently in common use is microfluidics. Microfluidics is a form of fluid handling that implements the mixing of organic solvents with an aqueous phase in a chip-based device [7]. The size of liposomes can be determined by different parameters like flow rate ratio, total flow rate, and concentration of organic solvent or lipid concentration [8]. The chips can be designed in different channel layouts like T- or Y-shaped [9], flow-focused [10] and droplet-based [11]. Some of the disadvantages of this method are the need to purify the samples from the used organic solvent [12], a high possibility of clogging of the channels due to the presence of proteins and lipids [13], and the low throughput of sample unless the method is upscaled [12]. Often, in combination with microfluidics, ionizable lipids are used. These are lipids that will form complexes that change their surface charge depending on the pH of their environment. Cationic ionizable lipids will remain neutral in a pH value of 7 but values under 7 will become cationic. The cationic nature aids in disrupting the membrane of the liposome and aids in the release of its cargo [14].

**Figure 1:**
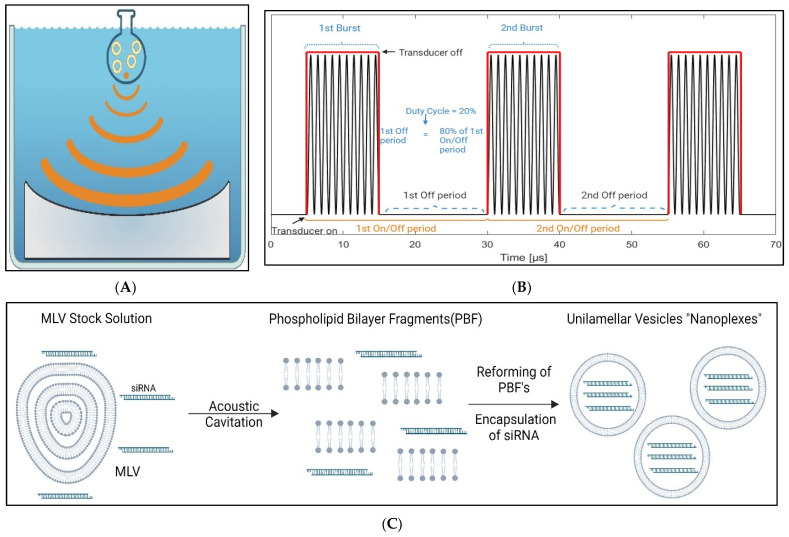
Schematic illustration of the AFU acoustic field during an ON period in the transducer, showing the focus of the AFU emitting from the concave surface of the transducer. Both the transducer and the sample are contained in a degassed thermostatic water bath (**A**). Schematic illustration of the AFU transducer output showing different optimized acoustics parameters (**B**). Proposed model for the formation of nanoplexes and siRNA entrapment. Ultrasound-induced cavitation breaks up lipid bilayers of MLVs into small PBFs, exposing their hydrophobic tails to the aqueous medium. To fence those parts back away from the medium, they quickly self-assemble and make large unilamellar vesicles that can entrap siRNA present in the aqueous medium, called “nanoplexes” (**C**).

New alternative methods are needed for versatile scalable production of lipoplexes in aqueous environments. Herein, we introduce adaptive focused ultrasound (AFU) for lipoplex generation. Cavitations with precise effect on nanoplex formation can be introduced to liposomes in a solution, thereby generating small vesicles and/or lipid bilayer fragments. Ultrasound is a technique that is used in diverse ways in medicine. It is used in clinical settings for imaging techniques due to its safety in vivo [15,16,17]. In focused ultrasound, the transducer is set up in a concave shape for the soundwaves to merge instead of spreading apart. Figure 1A illustrates the acoustic field of the AFU during an active sonication (ON period). It shows the focal point of the ultrasound emission originating from the concave surface of the transducer in a small glass vial that contains lipids and RNA molecules. The transducer and the sample are enclosed within a water bath, with thermal control, ensuring precise temperature control during experimentation. The principle of superposition offers a means to mitigate variable interferences arising from reflection by optimizing the shape of the transducer. This optimization aims to ensure that the bursts of ultrasound field emitted from the ultrasound source arrangement converge to a focal point, maximizing mechanical energy when cavitation threshold is exceeded. In this study, the commercially available AFU instrument utilizes a single-element transducer with a concave surface, which defines the volume and distance of the focal point from the transducer surface. The stability of the acoustic signal emitted by the transducer enables iterative feedback to the frequency of the electrical input, particularly near its central resonance frequency, to maximize the ultrasound transmission. This iterative process ensures maximal power transmission efficiency. Figure 1B illustrates the AFU transducer output at 10 cycles per burst with a 20% duty cycle. In the actual experiment, cycles per burst were 500, but the figure is just for illustrative purposes. We hypothesized that AFU breaks up the MLVs to create phospholipid bilayer fragments (PBFs) based on shear forces provided by acoustic cavitation. Thermodynamically unstable PBFs subsequently fuse and self-assemble to form thermodynamically stable unilamellar vesicles. During the process of reassembling, the therapeutic drugs are engulfed by the aqueous core of the newly formed particles, which we call nanoplexes. A visual principle of this hypothesis is shown in Figure 1C.

We need to explore methods that are not only effective but also feasible and scalable by creating more consistent lipid–nucleic acid complexes, a key advancement in lipid-based delivery systems. AFU-assisted nanoplexes offer the potential to make significant strides in RNA delivery as they are designed to be small and uniform with the capacity to protect therapeutic nucleic acids from systemic degradation. We continue exploring the designing and making of advanced nanoplexes that can contribute to advancements in RNA therapeutics.

## 2. Materials and Methods

### 2.1. Materials

N1-[2-((1S)-1-[(3-aminopropyl)amino]-4-[di(3-amino-propyl)amino]butylcarboxamido)ethyl]-3,4-di[oleyloxy]-benzamide (MVL5), 1,2-dioleoyl-sn-glycero-3-phosphocholine (DOPC), 3ß-[N-(N′,N′-dimethylaminoethane)-carbamoyl]cholesterol hydrochloride (DC-cholesterol), and 1,2-distearoyl-sn-glycero-3-phosphoethanolamine (DOPE) were from Avanti Polar Lipids (Alabaster, AL, USA). The CD44 siRNA (Eurogentec, 5′-> 3′: CGU-GGA-GAA-AAA-UGG-UCG-C55, 3′-> 5′: GCG-ACC-AUU-UUUCUC-CAC-G55) and control siRNA (Eurogentec, Seren, Belgium, SR-CL000-005) were used as a model for the complex preparation and transfection experiments (Eurogentec). Other chemicals were of analytical grade and from standard sources. Amounts of 5 mM HEPES (1 M, BP299-100, Thermo Fisher Scientific, Zaventem, Belgium) and 0.1 mM EDTA (0.5 mM, E8008, Sigma-Aldrich, Gillingham, UK) were used to make the liposome preparation buffer containing 5 mM HEPES and 0.1 mM EDTA.

### 2.2. Preparation of MLV Liposomes

Lipids DC-cholesterol:DOPE [18] and MVL5:DOPC [19] are often selected for preparing liposomes for transfection because of their unique properties that enhance the efficiency and stability of the liposomal formulations. We chose these formulations to test their efficacy and encapsulation efficiency under focused ultrasound to make nanoplexes. Appropriate amounts of the lipid stock solutions were mixed in chloroform to obtain the desired compositions. The solvent was removed under a stream of nitrogen, and the lipid residues were subsequently maintained at reduced pressure overnight to remove any trace of chloroform. The dry lipid film formed was then hydrated at 60 °C for 1 h in the previously described buffer, with stirring, leading to MLV formation.

### 2.3. Preparation of Nanoplexes Using Focused Ultrasound

**Figure 2:**
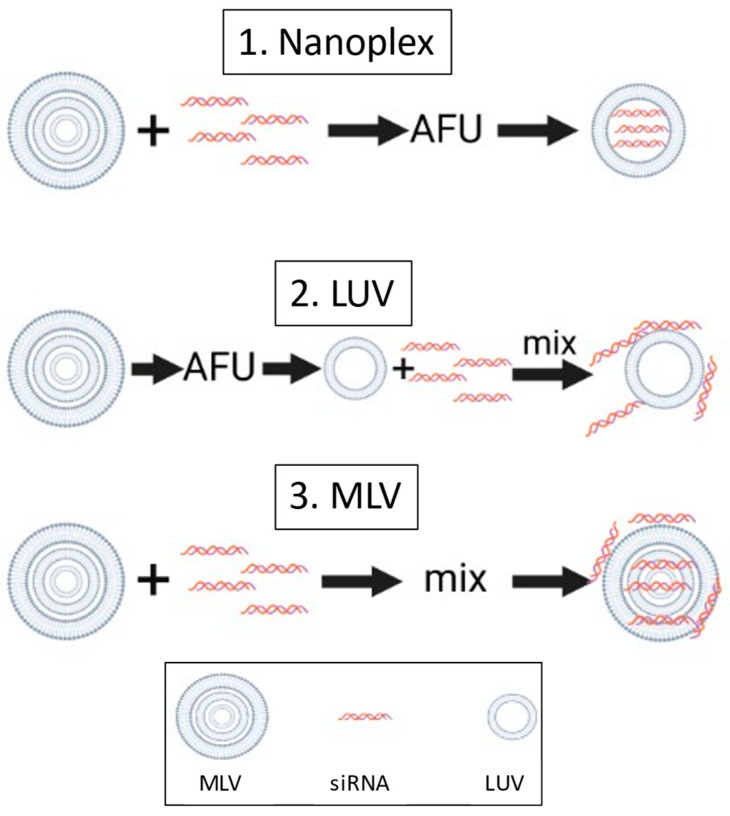
Schematic representation of the process for producing different complexes with lipid vesicles.

Since there was no prior art in making lipoplexes through focused ultrasound, in the beginning, it was more of an iterative process. Earlier we used this technique to make small unilamellar liposomes [20]. MLV formed with different lipid compositions together with siRNA molecules were subjected to acoustic energy using a commercial AFU sonicator (S2, Covaris, Inc., Woburn, MA, USA) to make unilamellar liposomes entrapping siRNA molecules to make nanoplexes. The final settings for AFU were set as follows. Intensity shows the amplitude of the waves that are created by the transducer. The duty cycle sets the transducer’s duration to create ultrasound bursts with a given duty cycle; the setting used here was 20%. Cycles per burst are the number of waves that are generated in each burst; this was set at 500. Treatment time for each sample was 10 min during 10 cycles, with each cycle set to 60 s. The AFU treatment temperature was kept at 20 degrees using the Haake C25/F6 recirculating water chiller unit (Haake, Thermo Fisher Scientific, Waltham, MA, USA). For every lipid formulation, 3 different forms of particles were prepared to test our hypothesis and the functionality in gene delivery of the resulting formulations. The different procedures are presented in Figure 2. Nanoplex particles were made by adding MLV stock solution with siRNA. AFU was then applied to test our proposed loading hypothesis of AFU, as shown in Figure 1C. LUV particles were made by applying AFU to MLV stock so that the reformation of PBFs happened before adding siRNA. The sample was then vortexed to mix the sample. The siRNA would then bind to the outside of the particles, respectively. MLV particles were made by adding siRNA to the MLV stock solution and vortexing for 30 s. Total lipid concentrations under focused ultrasound treatments were 0.2 mM for DC-cholesterol: DOPE (ratio 30:70) and 0.1 mM for MVL5: DOPC (ratio 40:60) liposome treatments. All formulations contained 200 nM of siRNA. This initial concentration was chosen to ensure that, upon dilution in the cell culture medium during transfection, the final siRNA concentration would be approximately 40 nM [18]. All samples were made in the Hepes buffer, as previously described.

### 2.4. Cell Culture

Two cell lines, human melanoma (MV3) and human retinal pigment epithelial (ARPE-19), were used in this study. MV3 melanoma cells are previously described by van Muijen et al. [21]. MV3 cells were maintained using Dulbecco’s Modified Eagle Medium (DMEM) with 4.5 g/L glucose (Lonza, 12-614F, Walkersville, MD, USA), 10% Fetal Bovine Serum (FBS) (Gibco, Paisley, UK), 1% L-glutamine (200 mM in 0.85% NaCl solution, Lonza, Verviers, Belgium), and 1% penicillin/streptomycin (10,000 U penicillin/streptomycin/mL, Lonza, Verviers, Belgium). Passaging was performed twice a week at a 1:15 ratio using 1× trypsin (0.5% trypsin (*w*/*v*) and 0.02% EDTA (*w*/*v*) (Biochrom AG, Berlin, Germany. A new cell batch was started after approximately 10 passages (1.5 months) of the current batch.

ARPE-19 cells are previously described by Hellinen et al. [22]. ARPE-19 cells were maintained using DMEM (Gibco BRL 31330-038, Bleiswijk, The Netherlands), 10% FBS (Gibco), and 1% penicillin/streptomycin (Lonza). Passaging was performed once a week at a 1:10 ratio using 1× trypsin (Biochrom AG) and 1× PBS (Corning, Corning, NY, USA). The medium was renewed 4 days after cells were passaged. A new cell batch was started after 30 passages were reached in the current batch. For transfection experiments, MV3 cells were seeded at a density of 120,000 cells/well in 6-well plates to reach 50% confluency after 24 h incubation at 37 °C. ARPE-19 cells were seeded at 200,000 cells/well to reach a similar density after 24 h. Cells were cultured at 37 °C under 5% CO_2_.

### 2.5. Transfection Experiments

Before treatments, the maintenance medium was replaced by a medium without serum and antibiotics (V = 800 μL for treatments, V = 1000 µL for cell control). Target cells without any liposome treatments and cells treated with liposomal formulations containing control siRNA were used as controls. The sample wells were the different liposome treatments containing human CD44. After adding 200 μL of samples to the wells, the cells were exposed to final siRNA concentration of 40 nM [23]. After addition of the lipoplexes, the cells were incubated for 5 h. After 5-h incubation, the medium was changed to 1 mL of maintenance medium, and cells were further incubated for 31 h before harvesting the cells and expression analyses.

### 2.6. RT-qPCR

After 36 h of incubation, cells were harvested using T9424 TRI Reagent^®^ (Sigma-Aldrich). RNA extraction was performed by following the TRI Reagent^®^ Kit protocol. Total RNA concentration was measured using NanoDrop ™ One/OneC Microvolume UV Spectrophotometer (Thermo Fisher Scientific). One μg of RNA was taken for complementary DNA (cDNA) synthesis. Synthesis of cDNA from RNA was performed using Verso TM cDNA synthesis kit following the manufacturer’s protocol (Thermo Fisher Scientific) and Personal Cycler –PCR thermal cycler (Biometra, Göttingen, Germany). After synthesis, the concentration of cDNA was diluted to 10 ng/μL for all samples by adding RNA’s free H_2_O before continuing to qPCR. For qPCR, the human primers were diluted to 10 pmol/μL. For normalization, ARPO was used as an endogenous control gene. Primer sequences are shown in Supplementary Figure S1. The qPCR detection was carried out by LightCycler^®^ 480 SYBR Green I Master (Roche, Basel, Switzerland) protocol. LightCycler^®^ 480 Instrument II performed the program. Raw data from the samples were normalized against endogenous control using the 2^−(DDCt)^ method, where DDC_t_ was calculated as follows: DCt(treatment) − DCt(vehicle) and DCt is Ct(target gene) − Ct(control gene).

### 2.7. Dynamic Light Scattering (DLS)

Hydrodynamic particle diameters (Z_av_) and polydispersity indices (PDI) of liposomes and lipoplexes were determined optically with dynamic light scattering at 25 °C (Zetasizer Nano ZS, Malvern Instruments Ltd., Malvern, UK). Z_av_ measurements were conducted using a refractive index of 1.330 for the HEPES/EDTA buffer and a viscosity of 0.8872. The data were collected with a backscatter setup at 173 degrees. In the current research work, each data point represents the mean of three independent measurements. Unless otherwise stated, lipid concentration was 100–300 μM.

### 2.8. Cryo-Electron Microscopy (Cryo-EM)

LUV, LUV + siRNA complex and nanoplexes were vitrified in a Leica EM GP device at 22 °C and 70% humidity on glow-discharged Quantifoil 2/2 holey carbon grid in liquid ethane. Cryo-EM data were collected at eBIC at the Diamond Light Source (Diamond Light Source Ltd., Oxfordhire, UK) on an Krios 300 kV TEM (FEI Co., Hillsboro, OR, USA) equipped with a Gatan post-GIF K2 Summit detector (Gatan, Inc., Pleasanton, CA, USA). The images were recorded with a Thermo-Fisher Falcon 3 detector operated in the linear mode at 57,000× nominal magnification. Dose: 12 e/Å2; sampling: 0.26 nm/pixel. Concentrations were 0.1 mM for MVL5: DOPC vesicles and 0.3 mM for DC-cholesterol: DOPE vesicles. The concentration of siRNA was 200 nM in each sample.

### 2.9. Atomic Force Microscopy (AFM)

AFM was used to obtain a 2D and 3D size and surface analysis and to give further strength to our hypothesis of encapsulation of nanoplexes. The samples were processed in the same way as cryo-EM samples. Dimension Icon (Bruker, Billerica, MA, USA) was used for AFM analysis. Mica Disc (AGAR Scientific, Rotherham, UK, AGF7013) was used as a substrate to immobilize samples before analysis. During measurements, AFM probes (ScanAsyst FLUID+, Bruker/ScanAsyst-air, Bruker) were used. Liquid measurements were set up by adding 30 µL of liposome treatments on top of freshly cleaved mica surfaces. It was then left for 10 min to immobilize. Analysis was performed using peak force tapping mode and ScanAsyst-fluid+ probes with a peak force of 2.0 nN. Air measurements were prepared by adding 60 µL of liposome treatment on top of freshly cleaved mica surfaces and left to immobilize for 10 min. Excess liquid was then removed, and the mica was left to dry in air at room temperature overnight before analysis. Analysis was performed using peak force tapping mode and ScanAsyst-air probes with a peak force of 2.0 nN. All images were processed using Nanoscope Analysis 1.9 software.

### 2.10. Cytotoxicity Assay

MTT method was used to check for potential cytotoxicity of the liposome formulations and particles. Cells were seeded in 96-well plates (MV3: 4000 cells/well, ARPE19: 6500 cells/well) and treated with liposomes similarly as for knockdown studies. Also, LUV without siRNA was added to the panel to check for potential cytotoxicity from the liposomal formulation. After a 36-h incubation time, 100 µL of MTT work solution (0.5 mg/mL, Sigma) was added to each well and incubated for 2 h. The formed formazan crystals were then dissolved with DMSO, and absorption of the samples was measured at 570 nm with Multiscan Skyhigh from Thermo Scientific. Experiments were performed in triplicate and the average was calculated after subtracting the background from each well. Cell viability was calculated as a percentage.

### 2.11. Encapsulation Assay

Quant-iT™ RiboGreen™ RNA assay kit was purchased from Thermo Fisher Scientific. Encapsulation of siRNA was calculated by following the manufacturer’s protocol. Briefly, rRNA standard solutions (1000, 500, 200, 100, 20, and 0 ng/mL) were prepared in 1× TE buffer. Lipoplexes and LUVs of both formulations were prepared as usual. In the 96-well plate, the samples were diluted with TE to obtain a theoretical siRNA concentration of 350 ng/mL (26 nM). Similarly, some samples were diluted with TE containing 2% Triton to break up the liposomes. Afterwards, 100 µL of diluted Ribogreen reagent was added to the wells and incubated in the dark for 5 min at room temperature. Fluorescence intensity was measured with TECAN infinite M200 (emission and excitation wavelengths at 485 and 528 nm). Emission from wells containing no Triton was seen as unencapsulated siRNA, whilst fluorescence intensity from wells containing Triton was seen as the total amount of siRNA. Concentrations were calculated by plotted standard curve and encapsulation efficiency was calculated.

### 2.12. Data Analysis

Microsoft Excel 2022 and IBM SPSS Statistics 27 software were used to quantify and analyze the results. Raw data from qPCR were first brought into Excel for quantification and analysis. Thereafter, the results were statistically analyzed with SPSS. *p*-values were indicated as significant by using of stars: no significance (NS), *p* < 0.05 *, *p* < 0.01 **, *p* < 0.001 ***.

## 3. Results

### 3.1. Formation of Lipid Complexes and Physicochemical Characterization

**Figure 3:**
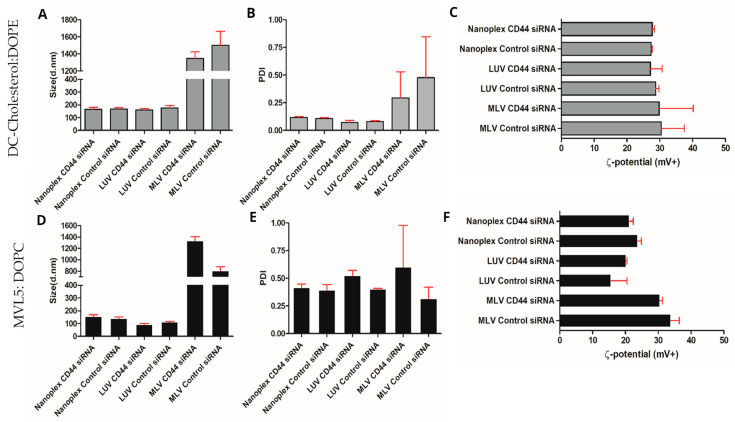
Physicochemical characterization of the lipid complexes developed in this study. Hydrodynamic diameter, PdI, and ζ potential of the complexes formed using DC-cholesterol: DOPE in HEPES buffer (**A**–**C**) and using MVL5: DOPC in HEPES buffer (**D**–**F**). Values are represented as the mean with standard deviation (s.d.) (*n* ≥ 3).

Size, PDI, and zeta (ζ) potential of lipid complexes are presented in Figure 3. Numerical values are reported in Supplementary Table S1. We also measured the size of original DC-cholesterol: DOPE formulation (without containing siRNA) after AFU irradiation, which was 135.5 nm, PDI 0.122, and zeta potential + 48.5 mV. The AFU parameters were optimized to generate nanoplexes with sizes around 100–200 nm (Figure 3, Supplementary Table S1). This is shown as evidence by the liposomes closely approaching the desired size range, as depicted in the “size distribution by volume” graphs. MLV-based simple complexation resulted in much larger lipoplexes and higher polydispersity (Figure 3A,D). DC-cholesterol: DOPE formulation and MVL5:DOPC formulation PDI’s of nanoplexes and LUVs were in the acceptable range of <0.5 [24] (Figure 3B,D). MLVs of both formulations were of µm size and showed bigger distribution in polydispersity, which was expected, since they did not receive AFU treatment so they would be bigger and more polydisperse compared with treated samples. The zeta potential of both formulations was positive, showing that all particles were cationic. The AFU parameters were optimized to generate nanoplexes with sizes ranging from 100 to 200 nm. The size, polydispersity index (PDI), and zeta (ζ) potential of the lipid complexes were measured and analyzed. The nanoplexes achieved this desired size range, as depicted in the “size distribution by volume” graphs (Figure 3). When the lipoplexes were formed using the MLV-based simple complexation method, larger lipoplexes and higher polydispersity were observed. This was expected as the MLV-based method did not involve AFU treatment, leading to larger and more polydisperse lipoplexes. The DC-cholesterol: DOPE formulation and MVL5: DOPC formulation showed PDI values of the nanoplexes and LUVs within the acceptable range of <0.5 (Figure 3B,E). This indicated a relatively narrow size distribution of the lipid complexes, suggesting their stability and uniformity. The MLVs of both formulations were of micrometer size and exhibited a larger distribution in polydispersity. This was consistent with the lack of AFU treatment, resulting in larger and more polydisperse complexes compared with the treated samples. The zeta potential of both formulations was positive. The difference in zeta potential between nanoplexes and MLVs, despite having the same lipid composition, can be attributed to structural differences. LUVs, with their single bilayer, had a more uniform and exposed surface charge, resulting in a lower zeta potential. In contrast, MLVs had multiple concentric bilayers, leading to higher surface charge density and cumulative effects from inter-lamellar interactions, thus exhibiting a higher zeta potential. The numerical values corresponding to these parameters are reported in Supplementary Table S1.

### 3.2. Cryo-EM and AFM Show Morphology of Nanoplexes and LUVs

**Figure 4:**
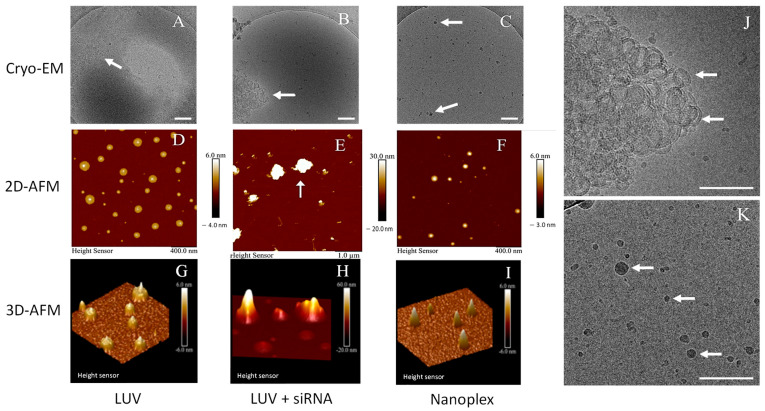
Cryo-EM and 2D and 3D AFM images of LUV(**A**,**D**,**G**), LUV + siRNA (**B**,**E**,**H**,**J**), and nanoplexes (**C**,**F**,**I**,**K**) of MVL5 formulation. Higher magnification images from panel (**B**,**C**) are shown in (**J**,**K**), respectively. White arrows in all panels point the morphology of different formulations. Scale bars 1 µm.

Cryo-EM imaging (Figure 4A–C) and 2D (Figure 4D–F) and 3D AFM air measurements (Figure 4G–I) showed that MVL5 formulations were spherical particles. DC-cholesterol formulation is shown in Supplementary Figure S2. MVL5 LUVs characterized by cryo-EM are shown in Figure 4A. When LUVs were mixed with siRNA, they agglomerated and formed bigger complexes, as evident in (Figure 4B,J). Interestingly, AFU-assisted nanoplexes clearly showed monodispersed particles with high electron density, suggesting the encapsulation of the nucleic acid siRNA (Figure 4C,K) The findings of cryo-EM were further supported by AFM imaging. The 2D AFM image (Figure 4D) and 3D AFM image (Figure 4G) of AFU-generated LUV showed a height of around 6 nm. The height of LUV + siRNA complexes was higher, up to 30 nm (Figure 4E,H). Further, nanoplexes 2D AFM (Figure 4F) and 3D AFM images (Figure 4I) showed complexes height of 6 nm, which correlated with AFU-assisted LUVs and cryo-EM characterization of nanoplexes. DC-cholesterol:DOPE complex characterization by cryo-EM and AFM is shown in the Supplementary Data (Figure S2). LUV generated after AFU treatment showed nanoparticles of around 100 nm when no siRNA was present (Figure S2A, white arrow), 2D AFM image (Figure S2D) and 3D AFM image (Figure S2G) of AFU-generated LUVs. When LUVs were mixed with siRNA (LUV + siRNA), they agglomerated and formed microcomplexes (Figure S2B). The 2D AFM images showed a random distribution of clustered complexes of size in micrometers (Figure S2E, white arrow). Moreover, a huge increase in cross-sectional height profiles was shown with 3D AFM, suggesting agglomeration (Figure S2H). AFU-assisted nanoplexes having siRNA were uniformly dispersed of size less than 100 nm, and a high electron density inside nanoplexes suggested the presence of siRNA (Figure S2C, white arrows). The 2D AFM images and 3D AFM images of nanoplexes showed the free distribution of nanoplexes (Figure S2F and Figure S2I, respectively).

### 3.3. Encapsulation Efficiency

**Figure 5:**
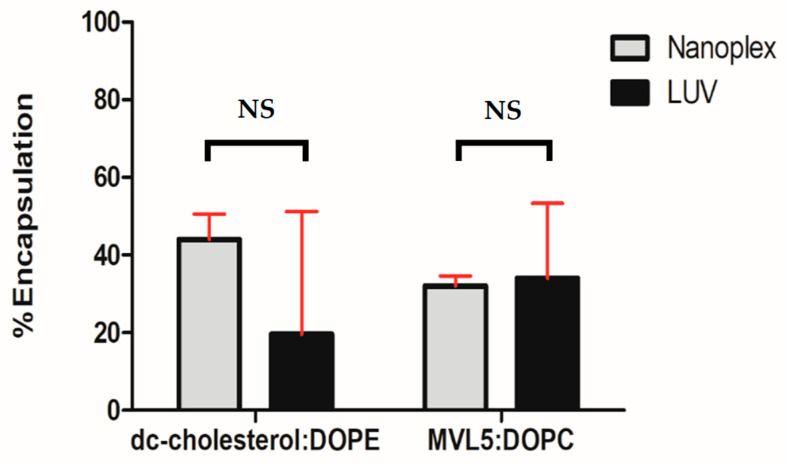
Encapsulation efficiency of nanoplexes and large unilamellar vesicles (LUVs). Independent sample *t*-test was used for both formulations. *n* = 3, NS = no significance.

The encapsulation assay of DC-cholesterol: DOPE formulation showed 44% of lipoplexes and 20% efficiency of siRNA encapsulation in LUV particles, respectively (Figure 5). The encapsulation efficiency of MVL5: DOPC formulation showed a similar level of efficiency, being 32% and 34% in nanoplexes and LUVs, respectively. Nanoplex encapsulation results showed less variability than LUV-derived lipoplexes for both formulations. This was somewhat expected since siRNA was added after AFU treatment.

### 3.4. siRNA Delivery Cytotoxicity of Complexes Developed in This Study

**Figure 6:**
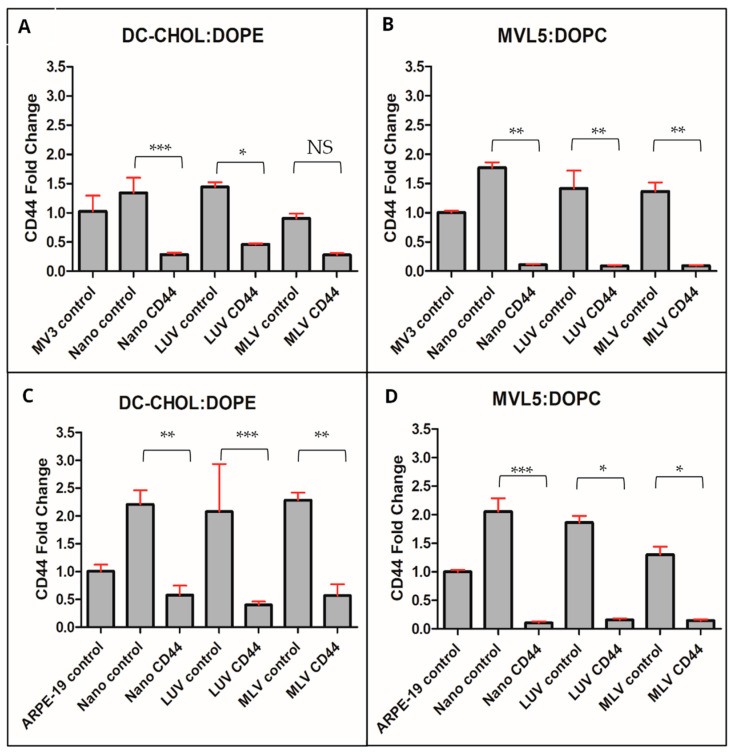
Results of CD44 knockdown experiments with qPCR of MV3 cells using DC-cholesterol (**A**) and MVL5 formulation (**B**). Bottom row shows gene knockdown results of ARPE-19 cells using DC-cholesterol (**C**) and MVL5 formulation (**D**). A non-parametric Kruskal–Wallis test was used in all samples. *n* = 4, normalized to housekeeping gene (ARPO), NS = no significance, * *p* < 0.05, ** *p* < 0.01, *** *p* < 0.001.

Transfections of MV3 (Figure 6A,B) and ARPE-19 cell lines (Figure 6C,D) were performed using complexes containing CD44-targeted siRNA, resulting in a successful reduction of CD44 expression. This result illustrates the effectiveness of utilizing nanoplexes formed through the method described in this study to achieve a stable delivery of siRNA. The nanocomplexes DC-cholesterol: DOPE and MVL5: DOPC exhibited a similar efficient knockdown of CD44 when compared with other complexes, indicating that the ultrasound treatment did not have any adverse effects on the stability of RNA molecules. The increased CD44 expression levels upon control siRNA treatments (Figure 6) were due to liposome-induced stress responses or liposome-plasma membrane interactions.

**Figure 7:**
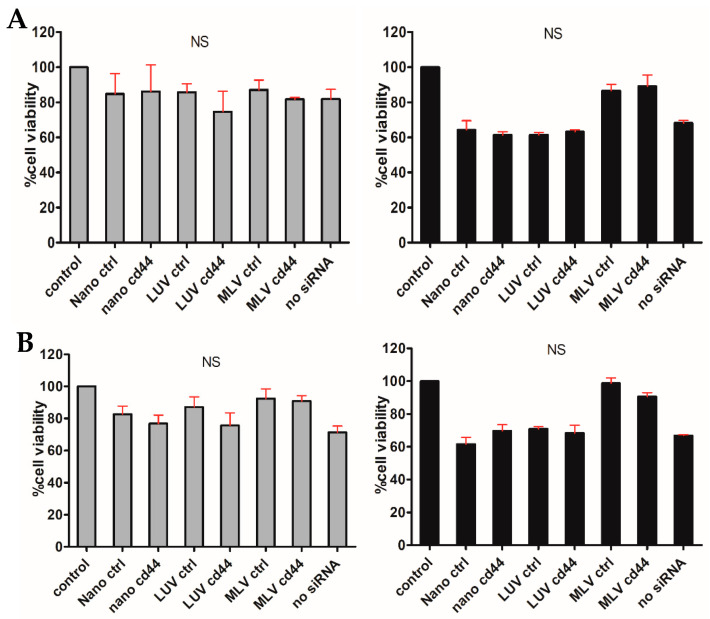
Cytotoxicity of different formulations measured by MTT assays in MV3 cells (**A**) and ARPE-19 cells (**B**). Grey columns show DC-cholesterol formulation (left panels) and black columns MVL5 formulation (right panels). Non-parametric Kruskal–Wallis test with Bonferroni correction was used for all samples. *n* = 3, NS = no significance.

An MTT assay was performed to test the in vitro cytotoxicity of the formulations in MV3 (Figure 7A) and ARPE-19 (Figure 7B) cells. No statistical significance was detected with any formulation in any of the cell lines. Cell viability was above 60 percent in all formulations with both cell lines.

## 4. Discussion

RNA interfering molecules such as siRNA have proven effective in certain conditions where gene-specific knockdown is desired. The main limitation of these molecules is their instability when administered in vivo and their inability to pass the cell membrane on their own, so they would need carriers like liposomes that help cancel out these problems to reach their full therapeutic potential [25]. Liposomes are highly versatile particles due to their ability to carry both hydrophilic and hydrophobic molecules and all modifications that can be applied to their surface to aid their targetability and increase circulation time in vivo [6]. Other lipid-based particles are already being applied in various treatments like “Onpattro” [26], and the most recent are the mRNA-vaccines against COVID-19 from Pfizer and Moderna that make use of LNPs as carriers [27,28].

There is a large variety of methods to prepare lipoplexes, but these preparation methods often do not limit the size and lamellarity of the particles, and extra processing techniques and solvents are needed to overcome these problems [29]. Processing methods like high-pressure extrusion and tip sonication are often used to make liposomes, but these techniques cannot be used to make lipoplexes. Also, the liposomes will come in contact with extra materials like filter membranes and titanium tips of sonicators that need to be put in the sample to make the process work. These procedures give possible points of contamination and impurities to remain in the solution and an extra sterilization step would be needed afterward. Techniques like extrusion also make it challenging to create multifunctional liposomes. The lipids and possibly used coatings could become stuck in the filter membrane, resulting in a mixture of coated and uncoated lipid particles [30]. This can give targetability problems if the primary function of the coating is to guide the particles to a particular place in the body. AFU can potentially overcome most of these problems because the sample does not come in contact with any extra materials like filter membranes, so the sample remains sterile, and the process is isothermal, so there is minimal fear of degradation of the molecules due to high temperatures [20,31]. This suggests the potential of the AFU-method to be optimized to make liposomal carrier particles that could be used in vivo.

In this study, the AFU method was used to create nanoplexes ranging in size from 100 to 200 nm. This size range has been described as the ideal size for liposomes to be used in vivo [32]. Nevertheless, there was still some polydispersity present. It has been insinuated that, for lipid nanoparticles, a PDI < 0.5 is seen as an acceptable threshold [33]. The FDA sets no recommendations regarding the PDI of liposome drug products [34]. The PDI of DC-cholesterol: DOPE nanoplexes and LUVs fell in this range. MVL5: DOPC liposomes showed to be more polydisperse but were still in range. MLVs of both formulations were in micrometer sizes and had a higher PDI with bigger deviations between measurements. This was expected since no processing by AFU was performed, so the sizes and dispersity of the particles can differ way more than nanoplexes and LUV+ siRNA complexes.

Focused ultrasound pulses of varying durations have been recently used to transport liposomes to the brain in a non-invasive manner [35,36,37]. AFU-assisted nanoplexes have great potential to transport a large variety of therapeutic agents, including hydrophilic and hydrophobic drugs, siRNA, antisense oligonucleotides (ASOs), but not bigger plasmid DNA because they can fragment under ultrasound treatment [38]. Previous studies have shown the ability of nanoplexes to carry model drugs into the inner ear and efficient delivery of targeted liposomes to auditory nerve cells. Nanoplexes have also been shown to efficiently target TrkB receptor-expressing cells in different cell lines [3,4,6].

In the current study, nanoplexes having silencer CD44 siRNA, which performs as a model therapeutic drug, have shown high knockdown (>90%) in different mammalian cell lines (melanoma cell line, MV3; and retinal pigment epithelial cell line, ARPE-19). The zeta potential is an essential factor of liposomes because it interprets the stability of particles in a solution [24]. Danaei et al. considered a zeta potential above +30 mV to be acceptable for cationic liposomes, as they repel each other enough and have a better stability [39]. The DC-cholesterol: DOPE particles seemed to be slightly under this threshold. This could be solved by increasing the cationic lipid ratio in the formulation. MVL5: DOPC particles had lower zeta potential than the DC-cholesterol formulation.

### Benefits of AFU in Making Nanoplexes

Multilamellar vesicles (MLVs) are typically formed during liposome preparation, by using methods like “lipid film hydration” or “reverse-phase evaporation”. These methods involve dissolving lipids in an organic solvent and dehydrating them, resulting in the creation of MLVs. In our previous work, the novel technique involving AFU for the generation of unilamellar vesicles was introduced. We developed lipid unilamellar vesicles and peptide-targeted unilamellar vesicles for a model drug delivery, representing an innovative approach in the field of liposome and vesicle formation [40]. Our current hypothesis builds on this success, suggesting that highly deterministic, localized, and controllable AFU can induce precise cavitations within lipid MLVs in a solution containing nucleic acids. Cavitation is an oscillatory phenomenon of gas bubbles in a liquid due to pressure gradients [20]. In this study, generated by ultrasound, the acoustic pressure waves from AFU were demonstrated to lead to the budding of smaller vesicles and the fragmentation of larger MLVs into PBFs. These PBFs then self-assembled into thermodynamically stable nanoliposomes, effectively entrapping therapeutic nucleic acids (such as RNA) present in the solution, referred to as “ultrasound-assisted lipid-based nanoplexes”. The AFU method offers several advantages. It is non-contact and essentially isothermal, allowing for low-temperature formulations. There is no material loss, and the process is highly controllable for desired nanoplex sizes. Co-solvents are not required, eliminating the risk of cross-contamination. Additionally, since it is a non-invasive method, the entire process can be conducted in sterile conditions. Furthermore, it is a speedy process and can be scaled up for large-scale production in a continuous flow setup. Furthermore, a wide range of lipid compositions can be used and combined with various payloads, including different targeting and imaging agents [20,40]. Even the nanoplexes and liposome complexes demonstrate more or less similar transfection efficiency in in vitro cell culture studies. Additional benefits or insights gained from the novel method beyond the improved particle shape are as follows: (a) better-encapsulated nanoplexes can provide a protective barrier against nucleases and other degradative enzymes present in biological fluids, potentially enhancing the stability and half-life of the siRNA; (b) encapsulated siRNAs can offer a more controlled and sustained release, which might be beneficial for maintaining therapeutic levels of siRNA over a longer period; (c) nanoplexes can be functionalized with targeting ligands [41,42]. Currently, several targeting conjugates, including antibodies, engineered proteins, peptides, aptamers, and lipids, are under development for siRNA extrahepatic delivery. Peptide conjugates are expected to expand as an siRNA delivery platform [43]. However, targeting ligands could also be added to lipoplexes, and the efficiency could be lower due to the potential dissociation of siRNA from the lipoplexes due to the hindrance from the targeting moieties. In a recent publication in PNAS, 2023, it was shown how the nanostructure of lipid nanoparticle RNA can be strategically manipulated to create highly efficient systems for RNA delivery. Their “cuboplex” nanostructures are significantly more efficacious at endosomal escape than traditional lipoplex constructs [44].

## 5. Conclusions

To conclude, our findings underline the suitability of AFU to create nanoplexes that can encapsulate therapeutic molecules in a sterile condition. This method has particular importance when designing nanoliposomes containing therapeutic nucleic acids for in vivo delivery. The results support our hypothesis that the created nanoplexes encapsulate the siRNA to prevent degradation. All complexes are shown to deliver the siRNA to the target cells, accomplished by efficient CD44 model gene knockdown. LUV + siRNA show agglomeration in both cryo-EM and AFM analysis. The presence of siRNA on the surface of LUVs in LUV + siRNA complexes is a potential threat to the degradation of therapeutic nucleic acid by endonucleases present in the target cells. AFU-assisted nanoplexes are a promising delivery vehicle as they can be made in a desired size range in sterile conditions with high encapsulation of therapeutic nucleic acids, as revealed by DLS, cryo-EM, and AFM analysis.

## Data Availability

Data is contained within the article.

## Supplementary Materials

The following supporting information can be downloaded at: https://www.mdpi.com/article/10.3390/nano14131089/s1, Figure S1: Primers used in the qPCR experiments; Table S1: Summary of the numerical values of size, PDI and zeta potential of different formulations; Figure S2: Cryo EM, 2D and 3D AFM of dc-cholesterol formulation. Scale bars in A–C: 1 µm.
