# Supplementary material for: Development of Focused Ultrasound-Assisted Nanoplexes for RNA Delivery
Source: Nanomaterials (Basel). 2024 Jun 25;14(13):1089. doi: 10.3390/nano14131089 (PMC11243722; doi:10.3390/nano14131089)
Supplement: Supplementary file 1 [file nanomaterials-14-01089-s001.zip › nanomaterials-3058963-supplementary.pdf]

Supplementary material

Gene name	Forward primer	Reversed primer
hARPO	5´ AGA TGC AGC AGA TCC GCA T 3´	5´ GTG GTG ATA CCT AAA GCC TG 3´
hCD44	5´ CAT CTA CCC CAG CAA CCC TA 3´	5´ CTG TCT GTG CTG TCG GTG AT 3´

Figure S1. Primers used in the qPCR experiments.

Table S1. Summary of the numerical values of size, PDI and zeta potential of different formulations.

DC-cholesterol:DOPE						
treatment	size(nm)	SD	PDI	SD	Zeta potential(MV)	SD
Nanoplex control siRNA	167,5	11,020	0,108	0,009	27,4	0,329
Nanoplex CD44 siRNA	165,4	15,059	0,116	0,010	27,4	0,694
LUV control siRNA	173,4	17,994	0,08	0,006	28,7	1,037
LUV CD44 siRNA	160,8	11,345	0,072	0,020	27,1	3,542
MLV control siRNA	1501	162,485	0,478	0,369	30,4	7,055
MLV CD44siRNA	1346	80,167	0,295	0,235	29,8	10,355

MVL5:DOPC						
treatment	size(nm)	SD	PDI	SD	Zeta potential(MV)	SD
Nanoplex control siRNA	140,9	10,839	0,393	0,049	23,7	1,087
Nanoplex CD44 siRNA	154,3	15,294	0,415	0,034	21,2	1,161
LUV control siRNA	111,2	6,363	0,404	0,004	15,6	4,888
LUV CD44 siRNA	95,1	5,931	0,526	0,046	20,2	0,294
MLV control siRNA	807	71,566	0,318	0,102	33,8	2,550
MLV CD44siRNA	1334	69,804	0,601	0,377	30,5	0,778

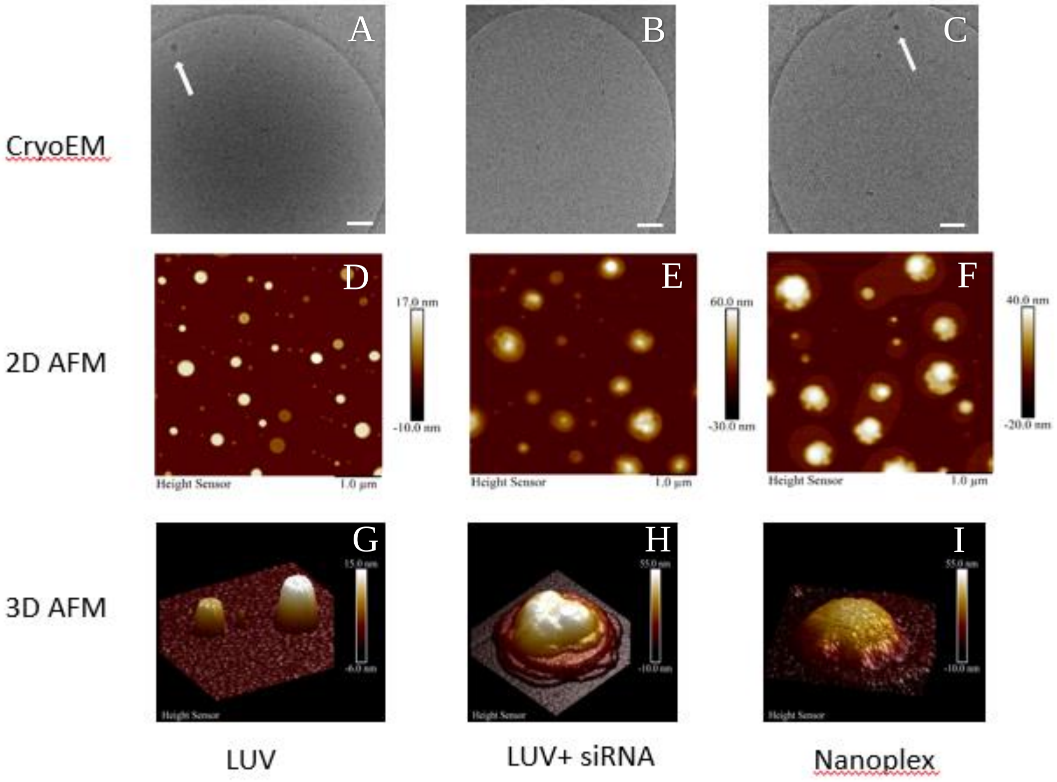

Figure S2 Cryo EM, 2D and 3D AFM of dc-cholesterol formulation. Scale bars in A-C: 1 μm.
